# Supplementary material for: “I just want to be normal”: Psychosocial experiences of adolescents and young adults with sickle cell disease in Kenya
Source: Glob Ment Health (Camb). 2026 Mar 6;13:e56. doi: 10.1017/gmh.2026.10175 (PMC13112317; doi:10.1017/gmh.2026.10175)
Supplement: Ochieng et al. supplementary material [file S2054425126101757sup001.zip › Appendix B_Focus Group Guides.docx]

**Appendix B : Focus Group Discussion Guides**

***Adolescents and Young Adult Focus group Discussion***

1. How does living with Sickle Cell Disease affect your health and happiness?
   - **Probe**: How does SCD affect your daily activities at home and school?
2. How do you feel about living with SCD?
   - **Probes:** Are there specific times when you feel more stressed or happy? What things make you happy or help you feel better when you're not feeling well? What changes have you experienced as you transition into teenagehood? Can you share experiences where you felt supported or not supported due to your condition?
3. What kind of support do you find most helpful in dealing with SCD?
   - **Probes**: What kind of people would you like to be supported by? As you transition into teenagehood, what specific support do you need? what kind of information to manage your condition would be helpful?
4. How do you communicate about your condition with friends and classmates?
   - **Probes:** How do they react? Do you feel like you can talk to them about it?
5. What kind of support do you receive at school and from the clinic to manage your SCD?
   - **Probes:** What additional support do you wish you had?
6. Can you describe your experience with SCD treatment and medication?
   - **Probes**: How do you feel about taking your medications? Are there any challenges with your medication routine or attending hospital/clinic visits?
7. How involved are you in decisions about your treatment and care?
   - **Probes:** Are there aspects of your treatment plan that you find confusing or challenging? What would make it easier for you to understand and manage your treatment?
8. If you could change anything about your treatment or support for SCD, what would it be?
   - **Probes**: What else can others (healthcare team, parents, community) do to be helpful?

***Health care Provider Focus Group***

1. From your perspective, what are the main challenges families face when caring for a child with SCD?

- **Probes:** What are some of the psychosocial problems that these families face? How do these challenges impact family dynamics and relationships? What strategies do you suggest to families for balancing caregiving responsibilities with personal needs?

1. How do you address the emotional and psychological needs of families caring for a child with SCD?

- **Probes**: What types of interventions or strategies have been helpful in the past? What has not been helpful? What support services or helpful strategies would you recommend for these families? How do you assist families in accessing psychological or counseling services? What else do you think needs to be done at the clinic or community levels?

1. What are your observations about families' experiences navigating healthcare for their child’s SCD treatment?

- **Probes**: What challenges have you noticed in their medical care and treatment access? How do you support families in managing medication accessibility and addressing financial challenges? What do you think the clinic can do better to support these families? How do you guide families in emergency situations like a sickle cell crisis? What do you think we can do to support these families in emergencies?

1. How do you ensure families are well-informed about SCD and its management?

- **Probes**: What educational materials or resources do you provide? How do you update families on new developments in SCD treatment? What has worked well in the past? What has not worked well? What else can be done? What platform, including digital platforms, can be used to keep most families informed?

1. What are your thoughts on the findings from the caregiver and child group discussions?

- **Probes**: How do these findings align with or differ from your professional experiences? What insights or changes in practice might you consider based on these findings?

***Caregivers Focus Group Discussion***

1. What are the main challenges you face in caring for a child with Sickle Cell Disease, and how does this affect your family?
   - **Probes**: How has your child’s condition affected your family relationships? How do you balance the demands of caring for your child with your personal needs and responsibilities?
2. Can you describe the financial and practical challenges you face in managing your child's SCD?
   - **Probes:** How do the costs of medications and treatments impact your family? In emergencies, such as a sickle cell crisis, how accessible is medical care?
3. Could you describe your experiences with navigating the healthcare system for your child’s SCD treatment?
   - **Probes**: What do you like about the services you receive from your clinic? What challenges or barriers have you encountered in accessing medical care and treatment? How easy or difficult is it to obtain the necessary medications for your child's treatment?
4. How has caring for a child with SCD affected your emotional well-being?
   - **Probes**: What kind of emotional, psychological, and social support have you found necessary or beneficial? Can you share your experiences in accessing psychological and/ or counseling services for yourself and your child?
5. What kind of support systems and resources do you use in caring for your child with SCD?
   - **Probes**: What kind of support do you receive from your family? Are you part of any SCD support groups? What do you like or dislike about the support groups? How has the support group impacted your experience? What kind of information would be helpful to manage your child’s condition? What improvements in support and resources would you like to see, both from healthcare providers and the community? What strategies or resources would you recommend to other caregivers dealing with the emotional challenges of caring for a child with SCD?
6. How do you engage with community awareness and education regarding Sickle Cell Disease?
   - **Probes**: What kind of community or societal support would make a significant difference in your life? How aware do you think your community is about SCD, and what awareness campaigns/education would be beneficial?
7. How do you stay informed about SCD and its management?
   - **Probes:** What type of information or education about SCD do you find helpful? What information is not helpful? What is the best way to give you information about SCD management, including digital platforms?  How adequately do you feel informed by health providers about new developments in SCD treatment?
