# Supplementary material for: “I just want to be normal”: Psychosocial experiences of adolescents and young adults with sickle cell disease in Kenya
Source: Glob Ment Health (Camb). 2026 Mar 6;13:e56. doi: 10.1017/gmh.2026.10175 (PMC13112317; doi:10.1017/gmh.2026.10175)
Supplement: Ochieng et al. supplementary material [file S2054425126101757sup001.zip › Appendix C_ TABLE 3_Adolescents_Psychosocial differences.docx]

**Table 2**

*Differences in psychosocial experiences of adolescents and young adulthood with sickle cell disease (SCD) across ecological levels and developmental stages.*

| **Ecological Level** | **Early Adolescence**  **(10-14 years)** | **Middle Adolescence**  **(15-17 years)** | **Late Adolescence/Young Adulthood**  **(18-25 years)** |
| --- | --- | --- | --- |
| **Individual** (Emotional & Psychological) | - Fear of immediate pain and activity limitations - Understands illness mainly through physical symptoms - Simple coping through distraction and prayer - Situational sense of being “different” during pain episodes - Simple coping through distraction and family-taught strategies - Desire to be “normal” like peers | - Emotional Distress from anticipating stigma - Internal shame and embarrassment about condition - Developing personal meaning about illness - Internal conflict between concealment and disclosure - Learning to manage frustration and embarrassment | - Existential concerns about mortality and future possibilities - Complex integration of illness into adult identity - Advanced coping through knowledge-seeking and reframing - Anxiety about independent disease management - Finding personal meaning and strength through suffering |
| **Social** (Interpersonal) | - Direct physical exclusion from peer activities - Complete dependence on family for care - Limited ability to explain the condition to others - School exclusion from physical activities - Little control over social interactions | - Strategic management of condition disclosure - Self-isolation to avoid negative peer perceptions - Difficulty explaining absences to school peers - Frustration with parental restrictions on activities - Awareness of being treated differently by teachers and peers | - Concerns about romantic relationships and partner acceptance - Navigating disclosure in workplace/education - Balancing dependency on caregivers with adult social roles |
| **Structural** (Healthcare & Systems) | - Healthcare mediated through caregivers - Limited knowledge about treatment rationales - Provider communication directed to parents | - Noticing problems in healthcare system - Desire for provider communication - Frustration with limited healthcare involvement - Beginning to question treatment approaches | - Assuming responsibility for healthcare navigation - Abrupt transition to adult services - Balancing education/work with health needs - Directly facing financial barriers - Growing awareness of systemic inequities in healthcare |
